# Enlarging pulmonary cyst: a rare form of alveolar adenoma

**DOI:** 10.1186/s13019-023-02409-9

**Published:** 2023-10-31

**Authors:** Yeoun Eun Sung, Mi Hyoung Moon

**Affiliations:** 1 Department of Hospital Pathology, Seoul St.Mary’s Hospital, College of Medicine, The Catholic University of Korea, Seoul, Republic of Korea; 2 Department of Thoracic and Cardiovascular Surgery, Seoul St.Mary’s Hospital, College of Medicine, The Catholic University of Korea, 222, Banpo-daero, Seocho-gu, Seoul, 06591 Republic of Korea

**Keywords:** Pulmonary cyst, Benign Neoplasm of lung, Alveolar adenoma

## Abstract

**Background:**

Alveolar adenoma is a rare benign tumour, usually presenting as a peripherally located solid mass, sometimes mimicking malignancy.

**Case presentation:**

A 37-year-old woman presented with chronic intermittent vague chest discomfort. The chest x-ray showed a simple cyst in the left lower lung field, and serial computed tomography (CT) over the following 2-year period showed rapid growth of the cyst, from 3.5 to 9.0 cm in diameter. The CT scan suggested bronchiolar communication, which was suspected to be the cause of growth, via check-valve mechanism. Thoracoscopic surgery was performed, and we found a thin-walled cyst in the lingular segment. Wedge resection was performed and the pathology was an unexpected alveolar adenoma which had grown on the terminal bronchiole, causing the alveolus to rupture and the cyst to grow. In 48 months of follow-up, there was no evidence of recurrence and the patient’s symptoms resolved.

**Conclusions:**

Rapidly growing pulmonary cysts can lead to complications including rupture with pneumothorax and haemothorax, and surgery is always indicated. Abnormally rapid growth may indicate an underlying pathology such as alveolar adenoma. Surgical resection is the treatment of choice and there have been no reported cases of recurrence. Here we present a rare form of alveolar adenoma, which was a form of rapidly growing pulmonary cyst.

## Introduction

Small pulmonary bullae are a common incidental finding, but large pulmonary cysts are rare and are usually discovered incidentally during routine check-ups or through imaging (chest x-ray or computed tomography [CT]) acquired for other reasons. Whether or not surgical resection is necessary for large pulmonary cysts depends on the number and characteristics of the cysts, the underlying pulmonary disease, and the presumed cause of the cysts.

The differential diagnosis includes congenital lung disease (i.e., cystic adenomatoid malformation or Birt-Hogg-Dubé syndrome), infectious disease, autoimmune disease, and malignancy [1]. Although definitive diagnosis depends on pathologic examination, the clinical impression hinges on the contours of the cyst wall, its size and overall characteristics, and patient presentation. Solitary cystic pulmonary lesions with thicker walls (> 15 mm) are at high risk (95%) of malignancy, whereas more thin-walled lesions (≤ 7 mm) are ordinarily benign [2, 3]. Both malignant and non-malignant pulmonary cysts can grow, but a rapidly growing cyst is a valid indication for surgical intervention [4].

## Case presentations

A 37-year-old woman was referred to our hospital after a health check-up at another hospital. She had an approximately 2-year history of vague chest discomfort and heaviness, and the chest x-ray from the referring hospital showed a cystic lesion in the left lower lung field. Abdominal ultrasound and routine blood tests results from the other hospital were all normal. She denied ever smoking and had no known comorbidities. Complete blood count with differential, blood chemistry, and electrocardiogram (ECG) were reviewed, and the results were all within normal limits.

**Fig. 1:**
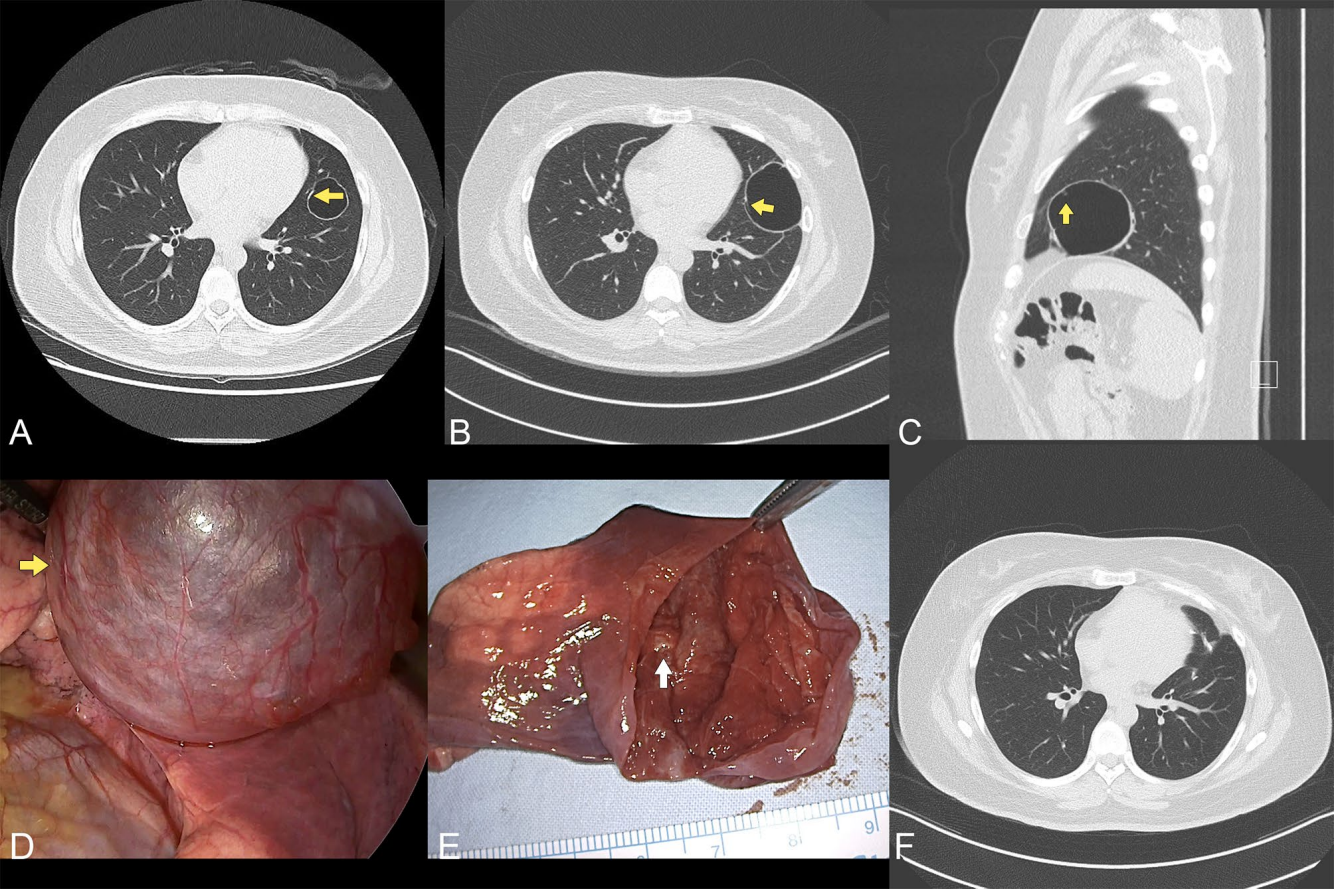
Diagnostic imaging and surgical findings: **(A)** initial chest CT demonstrating the 35-mm cyst; **(B, C)** axial and coronal views of cystic enlargement to 90 mm on subsequent CT. Yellow arrows indicate the suspected communication between cyst and bronchiole. **(D)** Intraoperative view of the cyst; **(E)** the opened cyst. The white arrow shows the terminal bronchiole, which has communication to the cyst. **(F)** Follow-up CT scan 2 years after surgery shows the healed surgical site

The cyst diameter on the initial chest x-ray was approximately 3 cm. The baseline low-dose chest CT showed a 35-mm intraparenchymal pulmonary cyst of the lingular segment (Fig. 1A). After a thorough discussion with the patient about the findings and possible diagnosis, surgical resection was deferred for a course of watchful waiting. At 12 months after the initial diagnosis, the CT scan showed that the cyst had grown from 3 to 9 cm. A bronchiolar connection to the cyst was also suspected at this time (Fig. 1B C). The patient was continuing to have intermittent chest discomfort but was otherwise stable. Considering the rate of growth of the cyst and the risk of rupture, after discussion with the patient, the decision was made for surgical resection of the cyst. As there was a risk of fistula formation with the suspected bronchial connection, it was decided to operate directly rather than attempt needle aspiration.

After general anaesthesia with double endotracheal intubation, the patient was placed on one-lung ventilation. Two-port video-assisted thoracoscopic surgery (VATS) was performed. On intraoperative inspection the thin-walled, broad-based cyst measured approximately 9 cm and was located in the lingular segment adjacent to the fissure (Fig. 1D and E). There was no visible parenchymal stalk. The cyst wall was so fragile that it ruptured during surgical manipulation, but without spilling fluid contents. The cyst was well-demarcated and removal, including the broad base of the cyst, was complete by wedge resection using endostaplers.

**Fig. 2:**
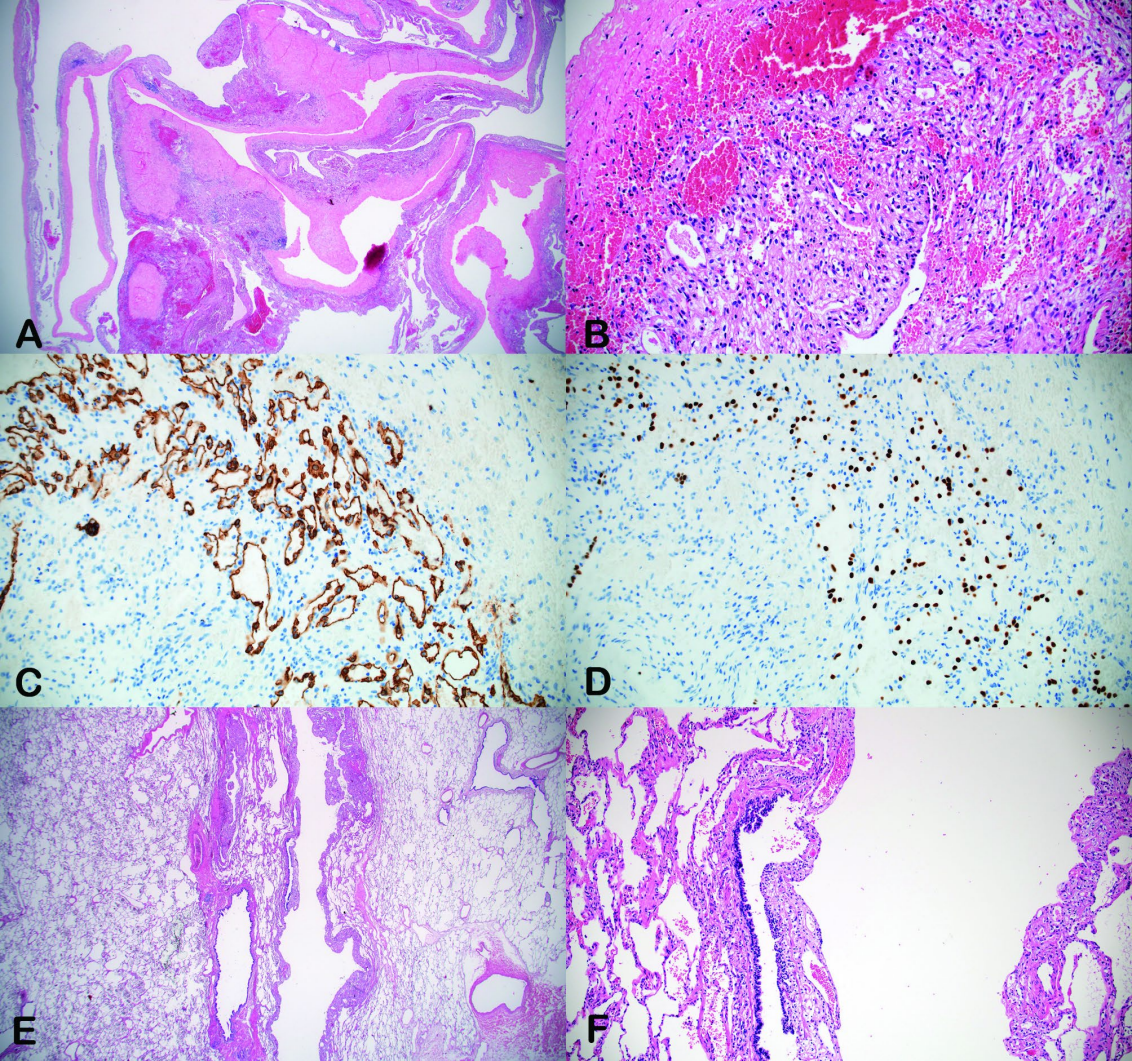
Microscopic appearance of the resected cyst: **(A)** low-power view (12.5×, haematoxylin and eosin (**H**&**E**) stain; **(B)** tiny compartmental spaces in cyst wall lined by bland, flattened to cuboidal cells (200×, **H**&**E**); **(C, D)** cellular positivity for pancytokeratin **(C)** and thyroid transcription factor-1 (TTF-1) **(D)** (200×); and **(E, F)** confirmed bronchiolar connection to the cystic lesion (12.5× and 100×, respectively; H&E stain)

On gross examination, the lesion was described as a unilocular cavity with an irregular wall with a maximum thickness of approximately 2 mm marked by fibrous thickening in the vicinity of the pleura (Fig. 2A). Microscopically, flat or cuboidal cells lined the tiny compartmental spaces incorporated within the cyst wall (Fig. 2B). Immunohistochemical staining indicated positivity for cellular expression of cytokeratin and thyroid transcription factor-1 (TTF-1), characteristic of type 2 pneumocytes (Fig. 2C and D). The type II pneumocytes showed bland appearing nuclei without atypia, excluding atypical adenomatoid hyperplasia or adenocarcinoma in situ. Cystic lesions such as Birt-Hogg-Dube syndrome or emphysema, which is characterized by usually diffuse lung change and surrounded by normal lung parenchyma, were also ruled out. The stromal cells were focally positive for smooth muscle actin and other mesenchymal markers (including S-100 and CD56a) but negative for HBM-45, a marker of melanocytic tumours. Based on the immunohistochemical staining results, other cystic pulmonary lesions including lymphangioleiomyomatosis could also be excluded. The bronchiolar connection tentatively detected by CT was verified under microscopy. The rapid growth of cyst was presumably attributed to a check-valve effect that caused the lesion to enlarge (Fig. 2E F). The final diagnosis was alveolar adenoma with cystic change, a rare benign tumour.

The patient’s postoperative course was uneventful. We removed a small Jackson-Pratt (JP) drain on postoperative day 1, and the patient was discharged on postoperative day 3. Given the rare but benign nature of the lesion, a brief multidisciplinary consultation with the pulmonologist, pathologist, and radiologist was called but no specific additional treatment besides regular follow-up was recommended. The patient remained symptom-free after surgery and there was no evidence of recurrence during a 2-year follow-up period (Fig. 1F).

## Discussion

Alveolar adenoma is a rare pulmonary tumour characterized by proliferation of alveolar epithelium and septal mesenchyme. It is considered benign, and accounts for < 1% of all lung tumours [5]. Roughly 33 published reports with 60 cases have appeared in the English medical literature [6, 7]. The first description was supplied by Yousem et al. in 1986 [8].

The cases reported in the English literature to date are summarised in Table 1. The average age was 52.9 years (ranging from 40 to 60) and there was a female predominance (21 male patients, 37 females, 2 not known). Most were asymptomatic and found incidentally during routine screening or evaluation of other conditions (29/60 patients). The most common symptoms were chronic cough (7 cases), either productive or non-productive, chest pain (5 cases), and mild dyspnoea (4 cases) [9–21]. Other non-specific symptoms, including generalized weakness, nausea, dysphagia, loss of appetite, and light-headedness, were also reported.

**Table 1:** Summary of reported cases of alveolar adenoma from the literature

No. of cases	Age	Sex	Sx	Radiologic findings	Location	Max diameter (cm)	Work up	Treatment	Follow-up (mo)	Recurrence	Other malignancy
6 [8]	45 74 54 58 64 59	F F F M M F	N Y N N N/A N	Solid nodule Solid nodule Solid nodule Solid nodule Solid nodule Solid nodule	LLL RML RUL LLL RUL RUL	2.0 2.5 2.5 1.5 1.2 1.3	CT, BRS (4)	Wedge Lobectomy Lobectomy Wedge Lobectomy Lobectomy	13 120 12 56 13	NED NED NED NED NED	Pituitary adenoma (1)
1 [21]	60	F	Y	Solid nodule	LUL	1.0	CT	Wedge			None
1 [32]	67	F	Y	Solid nodule	RML	2.8	CXR, unknown	Enucleation	3	NED	No comment
1 [12]	55	F	Y	Solid mass	RLL	4.0	CT, BRS	Segmentectomy	32	NED	None
1 [33]	52	F	N	Solid nodule	LLL	2.0	CT	Wedge	12	NED	No comment
17 [10]	45 58 50 39 41 52 41 54 64 58 45 68 46 NA 74 59 55	M M M M F F F F M M F M F N/A F N/A F	N/A N N N N N N N N/A N N N/A N Y Y N Y	N/A Solid nodule Solid lesion Solid nodule Solid nodule Solid nodule Solid nodule Solid nodule Solid nodule Solid nodule Solid nodule Solid nodule Solid nodule Shadow Solid nodule Solid nodule Solid mass	LUL LLL LLL RLL LLL LLL LLL RUL RUL LLL LLL LLL N/A Right RML RUL RLL	1.5 1.9 N/A 2.0 1.1 3.0 2.5 2.5 1.2 1.5 2.0 1.9 N/A 3.0 2.5 1.3 6.0	CT, BRS (5)	Lobectomy (2) Wedge or other resection (8) Others: unknown but lung resection	FU in 5 pts (2, 2, 5, 8, 13 yrs)	NED	Melanoma Acromegaly Leiomyoma
1 [16]	47	F	Y	Solid nodules Solid nodules Solid nodules	LLL RUL LLL	2.0 1.0 1.0	CT, MRI, BRS	Wedge (LLL)	15	NED	None
1 [13]	34	F	Y	Solid nodule	LUL	1.6	CT, BRS	Wedge	12	NED	No comment
1 [28]	51	F	Y	Solid mass with cystic lesion	RUL	3.4	CT, BRS	Sublobar resection	18		No comment
1 [24]	69	M	N	Solid and cystic lesion	RUL	3.5	CT, BRS	Wedge	13	NED	None
1 [34]	43	M	Y	Solid and cystic component	LLL	1.1	XR, CT, PET	Wedge, LNS	18	NED	None
2 [11]	62 54	M M	Y N	Solid nodule Solid nodule	LLL LLL	1.5 4.0	CXR, MRI CXR, No comment	Segmentectomy Wedge	22 32	NED NED	No comment
1 [31]	71	M	N	Solid nodule	RLL	1.7	CT	Segmentectomy			Prostate cancer
1 [30]	58	F	Y	Solid nodule	LUL	0.8	CT	Partial lung resection	3	NED	None
1 [18]	38	F	Y	Cystic mass	LUL	9.1	CT	Partial lung resection			No comment
1 [35]	61	F	N	Solid nodule	LUL	2.4	CT, PET	Segmentectomy	12	NED	None
1 [36]**	59	M	N	Incidental on pathology specimen	RUL	0.2	CT	Lobectomy			Lung cancer (SqCC)
1 [37]	42	F	N	Solid nodule	RLL	1.2	CT	Wedge	12	NED	No comment
1 [14]	54	F	Y	Solid nodule	LLL	1.8	CT, PET, Brain CT, Abdomen CT, BRS	Lobectomy	12	NED	Gastric cancer
2 [17]	24 35	M F	N Y	Solid nodule Solid mass	LLL RUL	1.8 5.0	CT, PET CT	Wedge Wedge	7 132	NED NED	No comment No comment
1 [9]	60	F	N	Solid nodule	RLL	7.0	CT, MRI	Wedge	6	NED	Cysts in breast, liver, kidney
1 [29]	48	F	N	Solid mass	RLL	4.0	CT, MRI, bronchoscopy	Lobectomy	48	NED	No comment
1 [27]	67	M	Y	Solid and cystic lesion	RLL	3.9	CT	Wedge			No comment
1 [38]	47	F	N	Round mass with air inclusions (“abscess”)	RLL	4.0	CT	Segmentectomy	52	NED	Cerebral AVM
1 [23]	83	M	N	Solid nodule	LUL	1.8	CT, PET, BRS	Segmentectomy	48	NED	No comment
1 [15]	40	M	Y	Solid mass	LLL	5.1	CT	Lobectomy	26	NED	No comment
1 [39]	48	F	N	Solid mass	LLL	3.5	CT	Lobectomy	60	NED	Bronchogenic cyst
4 [20]	36 51 38 59	M F F F	Y Y Y Y	Solid nodule Solid nodule Solid nodule Solid nodule	LLL RUL RUL LLL	2.6 1.8 1.3 2.0	CT, PET, BRS CT, BRS CT, RS CT, BRS	Resection Wedge Wedge Wedge	34 180 120 96	NED NED NED NED	No comment
1 [40]	59	F	Y	Ground glass nodule	RUL	2.4*(7.4)	CT, Bone scan, Brain CT	Lobectomy	36	NED	No comment
1 [22]	48	F	Y	Solid nodule	LUL	1.2	CT, PET	Wedge			Colon tubulovillous adenoma, Pituitary adenoma
1 [19]	26	F	Y	Multicystic bullae	LUL	3.2	CT	Lobectomy			No comment
1 [6]	63	M	N	Solid nodule	LLL	4.0	CT, BRS	Lobectomy	12	NED	No comment
1 [7]	52	F	N	Solid nodule	RUL	1.2	CT, PET	Wedge	36	NED	Incidental AAH

Otherwise unnspecified, CT or MRI indicates chest CT and chest MRI.

AAH: alveolar adenomatous hyperplasia; AVM: arteriovenous malformation; CT: computed tomography; F: female; FU: follow-up; LLL: left lower lobe; LNS: lymph node sampling; M: male; mo: months; NED: no evidence of disease; No.: Number; RML: right middle lobe; RUL: right upper lobe; SqCC: Squamous cell carcinoma; Sx: symptoms

* On histologic exam, the largest diameter of the ground-glass cystic nodule was 7.4 cm

The reason is unknown, but the most frequent location of the alveolar adenoma is the left lower lobe (23 cases), followed by the right upper lobe (15 cases), left upper lobe (9 cases), and right upper lobe (8 cases). The mean size of the tumour is 2.5 cm, ranging from 0.2 to 9.1 cm. The alveolar adenoma size itself in the present case was also 2 mm, although the total size of cyst was 9 cm. The CT findings were mostly of solid, round, well-circumscribed, homogeneous lesions of the peripheral lung; purely cystic features, as in our case, were reported in 2 cases, and solid lesions with cystic appearance in 4 cases.

According to Fujimoto et al., magnetic resonance imaging (MRI) may be productive in some cases for delineating cystic spaces with central fluid and thin-rim enhancement [16]. MRI is a much more sensitive means of defining cystic lesions than other imaging modalities. On T1- and T2-weighted images, alveolar adenomas display low- and high-level signal intensities, respectively. Cystic changes due to the check-valve effect created by partial connection between tumor and bronchiole are apparently rare.

Surgical resection has been performed in most cases for both diagnostic and therapeutic purposes after CT scan. Fluorine-18-fluorodeoxyglucose positron emission tomography (PET) has been performed in only a few cases, and in most of these there was no evidence of infection or malignancy in the suspicious nodule. Roshkovan et al. reported an SUVmax of 0.6 for a 12-mm solid nodule, which was only slightly above the lung parenchymal background [22]. Nosotti et al. reported an SUVmax of 1.06 for a 1.8-cm lesion [14]. Okada et al. have reported the only case so far of malignant transformation of an alveolar adenoma; the SUVmax was 1.4 for a 1.8-cm nodule [23].

The pathogenesis of such tumours remains unclear, but their origins from type II pneumocytes is the consensus view. There is indeed some evidence supporting a transition to type II pneumocytes by primitive mesenchymal cell precursors [10, 11]. Microsatellite abnormalities have been demonstrated in the epithelial component but not the mesenchymal component [24]. Diploid DNA pattern and non-balanced t(10;16) translocation have been reported in some cases [11]. Grossly, as in our case, these are well-demarcated subpleural or intraparenchymal lesions. Histologic preparations show ordinary cystic walls lined by flattened or cuboidal epithelial cells [6].

There have been other cases where tumour growth has occurred during follow-up before surgery [8, 24]. Although the exact size changes are not described in those reports, one of them was a case of solid mass with a cystic component that showed interval size increase over 15 years [24]. There are also reports of growth of solid nodules. Okada et al. reported an increase in size from 11 to 18 mm during a 15-month follow-up [23]. Generally, alveolar adenoma seems to be an indolent tumour, and the rapid growth in our case was attributed to the check valve mechanism.

Cyst formation is a well-known characteristic of some types of malignant tumours. In squamous cell carcinoma, the cavitation is related to the tumour necrosis and keratinization. In adenocarcinomas, cavitation can be attributed to the tumour necrosis or to a unidirectional check-valve mechanism [25, 26]. As has been well described by Hsieh et al., the unidirectional check valve mechanism appears to be initiated when a tumour begins to grow in the alveolar wall and then invades the non-cartilaginous bronchiole; formation of cysts which may gradually increase in size eventually occurs when the alveoli are ruptured by the unidirectional gas flow [27].

Because the clinical presentation is so imprecise, a diagnosis of alveolar adenoma is challenging to validate. Frozen sections or small biopsy specimens may closely resemble normal lung tissue [28]. At times, alveolar adenomas may simulate other pathology, including bronchioloalveolar carcinoma or other malignancies, atypical adenomatous hyperplasia, sclerosing hemangioma, hamartoma, papillary adenoma, congenital cystic adenomatoid malformation, or bronchial adenoma. Immunohistochemistry is quite helpful in this regard. Alveolar adenomas are positive for TTF-1, a transcription factor involved in the lung-specific expression of surfactant proteins [10], and surfactant proteins in particular, namely prosurfactant proteins B and C, are important markers. If positive, the likelihood of alveolar adenoma is heightened. Furthermore, alveolar adenoma is routinely negative for CC10 protein, a marker of Clara cells.

Efforts to distinguish alveolar adenoma are especially critical when presenting as a solitary peripheral nodule. The decision for surgery is made chiefly for diagnostic purposes. Progressive growth thus warrants surgical resection for tumour verification, ruling out malignancy [29, 30]. At present, alveolar adenomas are considered benign and prognostically inconsequential once completely resected. Even in extended follow-ups (up to 15 years), Kavas et al. encountered no recurrences [20].

It is interesting to note that although the exact relationship is not clear, there have been some cases of co-existence of alveolar adenoma with malignant diseases or other benign adenomas. Alveolar adenomas have been found incidentally in several patients during the evaluation of other conditions including as prostate carcinoma, colorectal carcinoma, and pituitary carcinoma [8, 22, 31]. It is noteworthy that malignant transformation was confirmed in one case; the alveolar adenoma was surrounded by an adenocarcinoma component and the border zone showed a transitional pattern [23]. This was the first case of alveolar adenoma with malignant transformation. Adenomatous alveolar hyperplasia has also been reported as an incidental finding besides alveolar adenoma [7]. Apart from the pulmonary pathology, multiple systemic cysts (liver and kidney) were present in one report, which would warrant a systemic evaluation in the presence of suspicious symptoms [22].

Many of the points above are exactly why we need a multidisciplinary discussion. As well described in the interdisciplinary paper by Roshkovan et al., there are a number of questions to be answered. Among these are: should biopsy be performed before surgery? Is systematic workup, including PET, necessary before surgery? How much surveillance is necessary before surgery? What surgery should be decided and how extensive should surgery be? And what type of postoperative follow-up is required? [22]. In this case, actually, there was no multidisciplinary discussion before surgery because no specific pulmonary nodules were observed on serial CT scans, and the cyst was thought to be simple cyst. Instead, we had a multidisciplinary discussion after surgery to determine the best course of postoperative follow-up and management.

In conclusion, alveolar adenoma is rare and considered benign. However, with the potential for gradual progression and a report of malignant transformation, the treatment of choice is complete surgical resection. No recurrences have been reported after curative surgery.

## Data Availability

All of the data and materials used can be found in our hospital’s database.
